# Risk factors, evaluation, and treatment advances for adverse distal aortic remodeling after type A aortic dissection repair: a systematic review

**DOI:** 10.1186/s13019-026-04626-4

**Published:** 2026-08-17

**Authors:** Zu-sen Zhang, Li-feng Li, Xin Li, Pei-ling Lu, Dong-lu Wang, Yun-fei Luo, Cheng-lin Li, Xin Liu, Hong-shu Wang, Chong-bin Zhong

**Affiliations:** Cardiovascular Surgery, Chuxiong Yi Autonomous Prefecture People’s Hospital, Chuxiong, 675000 China

**Keywords:** Type A aortic dissection, Adverse distal aortic remodeling, Risk factors, Endovascular intervention, Systematic review

## Abstract

**Objective:**

Adverse distal aortic remodeling after type A aortic dissection (TAAD) repair is the principal cause of late aneurysm formation, rupture, and reintervention, significantly compromising long-term survival in patients with TAAD.

**Methods:**

This study is a systematic review. A comprehensive search of Chinese and English databases up to December 10, 2025 was performed to systematically identify imaging-based risk factors for adverse distal aortic remodeling after TAAD repair and to summarize the efficacy and limitations of current endovascular intervention strategies.

**Results:**

After systematically searching CNKI, Wanfang Data, VIP, PubMed, Embase, the Cochrane Library, and Web of Science, a total of 31 studies involving 5330 patients were included.

**Conclusion:**

Imaging-based risk factors for adverse distal aortic remodeling include early postoperative descending aortic diameter ≥ 40 mm, a patent or partially thrombosed false lumen, and distal anastomotic new entry (DANE). Endovascular techniques, including false lumen occlusion, aortic true lumen stenting, and adjunctive intimal fenestration, can effectively improve patient outcomes. Early identification of high-risk patients and individualized intervention are key to enhancing the long-term prognosis of individuals with TAAD.

## Introduction

Acute type A aortic dissection (TAAD) is a catastrophic aortic surgical disease. Despite continuous advances in surgical techniques and postoperative management, and although proximal repair strategies such as the frozen elephant trunk (FET) procedure have significantly reduced short-term mortality, persistent false lumen perfusion and degenerative changes of the aortic wall result in distal aortic disease that remains critical to long-term survival. Studies have shown that the 5-year survival rate after TAAD repair is only 55%–85%, and the development of post-dissection thoracoabdominal aortic aneurysm leading to aortic rupture in the chronic phase is the leading cause of late death among survivors after TAAD repair [1]. Notably, this patient population is younger than those with degenerative aneurysms, and the aorta remains in a dynamic remodeling process throughout life, posing even more formidable challenges for the management of aortic disease [2].

Current postoperative surveillance after TAAD is largely limited to maximal aortic diameter and expansion rate, and the indications for intervention are primarily based on a diameter > 5.5 cm or an expansion rate > 10 mm/year. Systematic understanding is lacking regarding residual dissection evolution, new entry tears, branch artery involvement, the true-to-false lumen ratio, and reintervention modalities [3]. Therefore, this study systematically reviews the high-risk imaging features of adverse distal aortic remodeling after TAAD repair and the endovascular management strategies, providing a reference for clinical risk stratification and endovascular intervention.

## Methods

### Literature search strategy

Two investigators independently conducted a comprehensive search of the following databases: CNKI, Wanfang Data, VIP Chinese Science and Technology Periodical Database, PubMed, Embase, the Cochrane Library, and Web of Science. The search was restricted to studies published in Chinese and English. The English search terms were as follows: (“Aortic Dissection“[Mesh] OR “Aortic Aneurysm, Thoracic“[Mesh] OR “aortic dissection*” OR “type A aortic dissection*” OR TAAD) AND (“Postoperative Period“[Mesh] OR “Postoperative Complications“[Mesh] OR postoperat* OR postsurg* OR after surg* OR post-surg*) AND (“False Aneurysm“[Mesh] OR “false lumen*” OR “persistent false lumen*” OR “patent false lumen*” OR “residual false lumen*” OR “distal aorta*” OR “aortic remodel*” OR “aorta remodel*”) AND (“Reoperation“[Mesh] OR “Intervention“[Mesh] OR reinterven* OR re-interven* OR reoperat* OR re-operat* OR “secondary intervention*” OR “reintervention” OR “treatment failure” OR “endoleak”) AND (“Disease Progression“[Mesh] OR “Prognosis“[Mesh] OR “Treatment Outcome“[Mesh] OR “Mortality“[Mesh] OR “Survival Rate“[Mesh] OR progress* OR prognos* OR outcome* OR mortalit* OR survival OR “adverse event*” OR remodel*). The Chinese search strategy, taking CNKI as an example, was: (title=Stanford type A aortic dissection OR aortic dissection type A) AND (title=adverse distal aortic remodeling OR dissecting aneurysm OR false lumen expansion OR imaging) OR (title/abstract=reintervention OR endovascular intervention).

### Inclusion and exclusion criteria

Inclusion criteria: ① Patients with Stanford type A aortic dissection who had at least one year of postoperative follow-up and at least one CTA examination reporting aortic diameter, false lumen diameter, intimal tears, residual branch arteries, and false lumen thrombosis. ② Studies on endovascular reintervention methods after type A aortic dissection repair.

Exclusion criteria: ① Duplicate studies; ② Animal experiments; ③ Literature reviews; ④ Full text unavailable, incomplete data, or erroneous research methodology.

### Data extraction

Data were independently extracted by two investigators, and any disagreements were resolved by a third investigator.

Data extraction items included: ① Authors, year of publication, study type, sample size, follow-up duration. ② Death, survival, aortic-related adverse events (aortic rupture, aortic-related death, aortic-related reintervention). ③ Aortic true lumen diameter, false lumen diameter, aortic diameter, annual aortic expansion rate, intimal tears (pre-existing, new). ④ Endovascular reintervention methods.

Given the significant heterogeneity among studies, no meta-analysis was performed. The Newcastle-Ottawa Scale (for non-randomized cohort studies) was used to assess study quality across three dimensions: selection of study subjects, comparability of groups, and outcome assessment. Studies scoring ≥ 6 were considered high quality. Because of significant heterogeneity among the included studies, meta-analysis was not conducted. This study did not involve patient data and was approved by the Ethics Committee of Chuxiong Prefecture People’s Hospital, which waived the requirement for ethical review.

## Results

### Search results and quality assessment

**Fig. 1:**
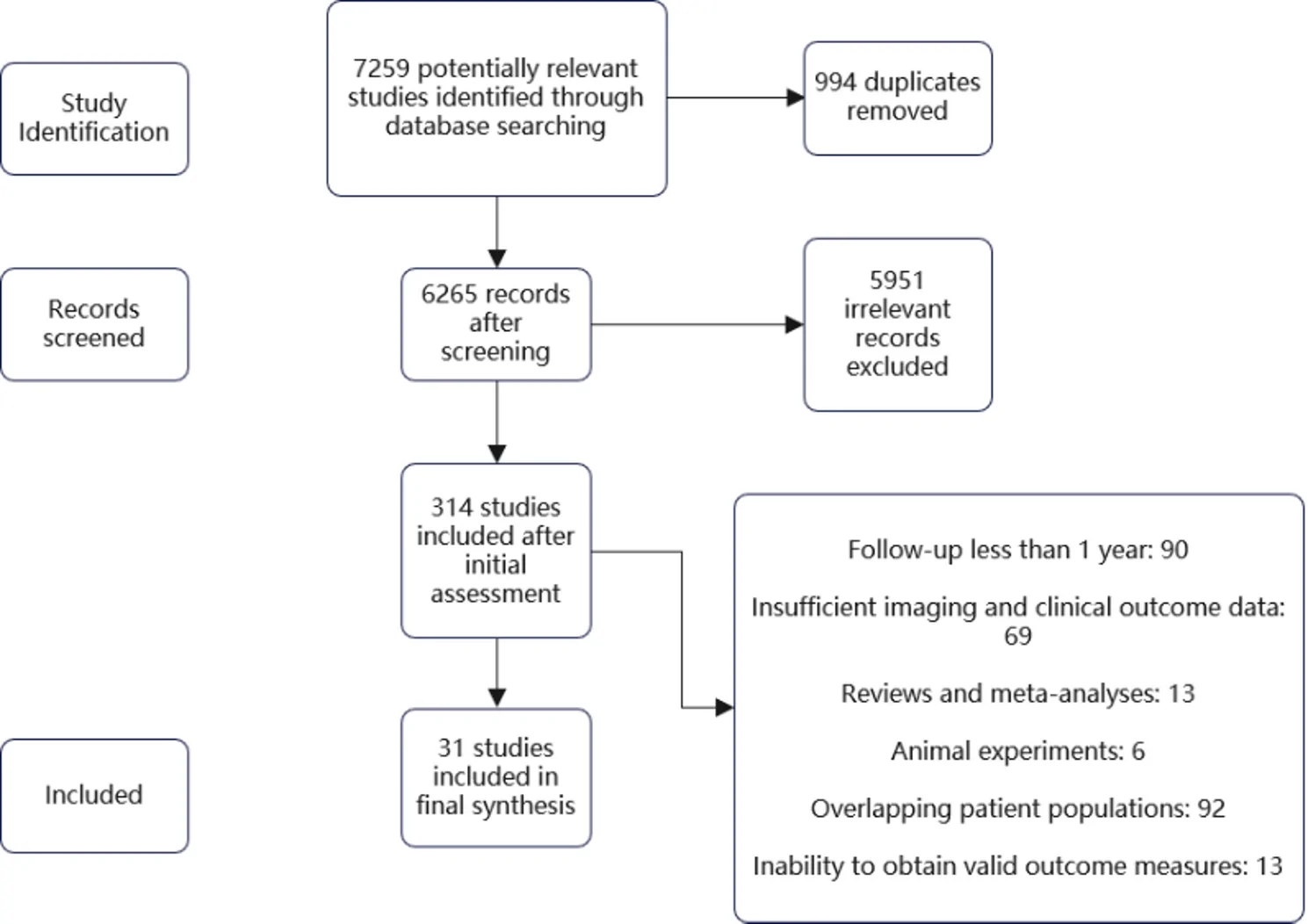
Study flow diagram

The initial search yielded 7259 relevant studies, and after removing duplicates, 6265 studies remained. Following screening, 31 studies were ultimately included, one of which was a prospective non-randomized cohort study, encompassing a total of 5330 patients (Fig. 1). Quality assessment showed that only 2.9% of the included studies had a score < 6. However, the majority of the included studies were single-arm studies, which precluded effective quality assessment. Among them, two studies employed propensity score matching to control for confounding factors. To avoid selection bias, low-quality studies were not excluded.

### Imaging-related risk factors

#### Aortic morphological parameters: diameter and expansion rate

Among the numerous imaging parameters, aortic diameter and aortic expansion rate are the two most thoroughly investigated morphological predictors with the most consistent evidence. However, the threshold for defining a hazardous diameter varies across studies—Zierer et al. stratified patients by 35 mm and 49 mm, Kimura et al. used 45 mm as the cutoff, and Leontyev et al. adopted 40 mm [3, 4, 6, 10]. Such discrepancies may stem from differences in study populations, measurement methods (traditional manual diameter measurement vs. semi-automated volumetric analysis), and follow-up durations; nevertheless, 40–45 mm has been widely accepted as the clinical alert threshold. Most studies have focused on the descending or thoracic aorta, whereas Biancari et al. identified preoperative maximal abdominal aortic diameter as an independent predictor of distal aortic reoperation (SHR = 1.041 per 1 mm increase, *P* < 0.001); when the abdominal aortic diameter exceeded 30 mm, the 10-year cumulative incidence of distal reoperation was significantly higher (12.0% vs. 5.7%, SHR = 2.076 at a threshold of 30 mm) [8]. This suggests that different aortic segments may require different risk assessment thresholds. Despite the heterogeneity, the conclusion that a larger aortic diameter and rapid expansion are independent predictors of reintervention is highly consistent across all studies (Table 1).

A total of four studies reported aortic growth rates [4, 5, 11, 12]. The reported aortic expansion rates varied considerably across studies, reflecting marked heterogeneity. Aortic growth may manifest as a slow, linear process or as rapid, segmental expansion; this variation may be related to differences in study methodology, the definition of growth rate, and the aortic segment assessed. The abdominal aorta represents a high-risk segment: several studies suggest that the expansion rate of the abdominal aorta may be no slower, or may even be faster, than that of the thoracic aorta [4, 12, 13]. Despite the discrepancies in absolute growth rate values, all studies consistently agree that a larger postoperative aortic diameter, a patent false lumen, and partial thrombosis are key factors predicting more rapid future expansion [4, 12] (Table 2).

**Table 1:** Studies on aortic diameter

Study	Sample size	Study design	Statistical adjustment method	Primary morphological predictor	Multivariate statistical results	Replicability in independent cohorts
Zierer, 2007	201 patients (168 for reintervention analysis; 69 for growth analysis)	Single-center retrospective cohort (1984–2006)	Multivariable Cox regression (29 variables, stepwise backward elimination)	Descending aortic diameter > 40 mm	*P* < 0.001; diameter < 35 mm → 11% growth, 35–49 mm → 22%, > 49 mm → 37%	Replicated in multiple subsequent studies (Leontyev 2017, Suzuki 2018, etc.)
Halstead, 2007	179 patients (89 for growth analysis)	Single-center retrospective cohort (1986–2003)	Multivariable Cox regression; hierarchical linear model	Initial diameter > 4 cm; diameter < 4 cm + patent false lumen	Initial > 4 cm: *P* = 0.005; patent false lumen: *P* = 0.004	Consistent with conclusions of Zierer et al.
Kimura, 2015	534 patients (451 analyzed)	Single-center retrospective cohort (1990–2013)	Multivariable Cox proportional hazards (stepwise method)	Distal aortic diameter > 45 mm	HR 4.4, 95% CI 2.51–7.62, *P* < 0.001	Consistent with the 40–45 mm threshold identified by Leontyev et al.
Suzuki, 2018	310 patients (278 analyzed)	Single-center retrospective cohort (2004–2017)	Multivariable Cox regression (stepwise forward selection)	Descending aortic diameter > 40 mm	Diameter: HR 1.12 per mm, *P* < 0.001	Validated 40 mm as the critical threshold
Leontyev, 2017	477 patients (105 analyzed)	Single-center retrospective cohort (1995–2012)	Multivariable logistic regression	Postoperative descending aortic diameter > 40 mm	OR 5.6, 95% CI 1.6–19, *P* = 0.006 (for diameter increase ≥ 10 mm)	Validated 40 mm as the critical threshold
Biancari, 2024	990 patients	Multicenter retrospective registry study (17 centers in 8 European countries, 2005–2021)	Multivariable competing risk analysis (Fine-Gray test)	Maximal abdominal aortic diameter (continuous); abdominal aortic diameter > 30 mm	SHR 1.041 per mm, *P* < 0.001; >30 mm group: 10-year reintervention 12.0% vs. 5.7% (adjusted SHR 2.076)	Multicenter validation, extending the predictive scope to the abdominal aorta

**Table 2:** Studies on growth rate

Study	Aortic arch (mm/year)	Descending/thoracic aorta (mm/year)	Abdominal aorta (mm/year)	Key findings
Halstead, 2007	0.8	1	0.8	Growth is slow and linear; patients with initial diameter > 4 cm or initial diameter < 4 cm but a patent false lumen experienced faster descending aortic growth.
Zierer, 2007	Not explicitly provided	Mean annual growth rate of 5.3 ± 4.5 during the follow-up intervals in which growth occurred	Not explicitly provided	The onset of growth is unpredictable (mean 59 months postoperatively); a larger aortic diameter is associated with a higher risk of growth.
Leontyev, 2017	Not explicitly provided	14.3% of patients had a diameter increase > 10 mm during follow-up	Not explicitly provided	Postoperative descending aortic diameter > 40 mm and a patent false lumen are independent predictors of a diameter increase > 10 mm.
Mittal, 2025	1.5 (0.1–3.9)	Upper segment: 2.8 (0.8–6.7)	3.1 (1.6–5.4)	The abdominal aorta exhibited the fastest growth; a patent false lumen was the only factor significantly associated with growth in the lower thoracic and abdominal aorta.
		Lower segment: 2.9 (1.2–6.0)		

#### False lumen status

A total of eight studies reported the relationship between aortic false lumen status and long-term prognosis [14–21]. False lumen patency is closely associated with distal aortic expansion, reintervention rate, and long-term mortality; however, the magnitude and mechanisms by which different false lumen states (completely patent, partially thrombosed, completely thrombosed) influence reintervention risk remain controversial. In the study by Kimura N et al., the partially thrombosed and completely patent states were combined into a “non-completely thrombosed” group for analysis, whereas Song SW et al. and Tsai MT et al. treated partially thrombosed as an independent category [19, 22]. Such differences in definition may render results not directly comparable (see Table 3 for details).

In studies comparing different surgical approaches, the series employing the frozen elephant trunk (FET) technique (e.g., Wada T et al.) reported a lower reintervention rate (89.9% freedom from reintervention at 5 years), whereas studies involving conventional ascending aorta or hemiarch replacement reported higher reintervention rates [23]. This suggests that the surgical strategy itself may be an important factor influencing false lumen outcomes.

Furthermore, follow-up duration ranged from a mean of 2.9 years to a median of 8.4 years; as reintervention events often occur in the long term, short-term follow-up may underestimate the true reintervention rate. Several studies (Vendramin I et al., Larsson M et al.) specifically investigated the effect of anticoagulation therapy on false lumen status [18], finding that although anticoagulation increases false lumen patency, it does not increase the risk of reintervention—a conclusion that diverges from the finding of Song SW et al. (2011) that early anticoagulation promotes false lumen thrombosis [24].

**Table 3:** False lumen status and expansion rate

Study (First author, year)	Sample size	Study design	Adjustment method	Statistical significance after multivariable analysis	Main findings (reintervention-related)	Inter-study heterogeneity / Replicability
Song SW et al. (2010)	118 patients (66 with adequate imaging data)	Single-center retrospective cohort study	Multivariable Cox regression analysis	Significant (*P* = 0.002)	Partial thrombosis is an independent predictor of aortic-related reintervention (*P* = 0.002); the partially thrombosed group had the highest 10-year reintervention rate.	Consistent with the findings of Tsai TT et al. (2007) in type B dissection; however, some studies consider patent false lumen, rather than partial thrombosis, as the primary risk factor.
Tsai MT et al. (2014)	67 patients	Single-center retrospective cohort study	Kaplan-Meier survival analysis, ANOVA	Significant (*P* = 0.013)	In the proximal descending thoracic aorta, the 10-year freedom from reintervention was 62% in the partially thrombosed group, 100% in the completely thrombosed group, and 88% in the completely patent group (*P* = 0.013).	Consistent with Song SW et al. (2010); however, Tsai TT et al. (2007) found that in type B dissection, partial thrombosis was associated with mortality rather than reintervention.
Kimura N et al. (2015)	472 patients (451 with CT data)	Single-center retrospective cohort study	Multivariable Cox proportional hazards regression	Significant (*P* = 0.001)	Patent false lumen is an independent predictor of distal aortic events (HR = 4.11, *P* = 0.001); 62% of patients had a patent false lumen postoperatively.	Consistent with most studies; however, this study grouped partial thrombosis with the patent group, which differs from the definitions used in other studies.
Mittal A et al. (2025)	50 patients	Single-center retrospective cohort study	Kruskal-Wallis H test, multivariable analysis	Significant (*P* < 0.05)	Patent false lumen was the only factor significantly associated with growth of the lower descending thoracic and abdominal aorta (*P* < 0.05); 2 patients with partial thrombosis required reintervention.	Small sample size, but results are consistent with Song SW et al. (2010).
Wada T et al. (2022)	196 patients	Single-center retrospective cohort study	Kaplan-Meier analysis	—	The 5-year freedom from distal aortic reintervention was 89.9%; FET promotes descending aortic remodeling, but its effect on remodeling beyond the stent graft is limited.	Lower reintervention rate compared to series not using FET; suggests that surgical strategy influences false lumen outcomes.
Katsuhiko Oda et al. (2025)	192 patients	Single-center retrospective cohort study	Univariable and multivariable analysis	Significant (*P* = 0.015/0.038)	In the patent false lumen group, more pronounced expansion was observed in the proximal (5.3 vs. 3.25 mm, *P* = 0.015) and distal (3.1 vs. 1.9 mm, *P* = 0.038) descending thoracic aorta; there was no significant difference in reintervention rate between groups (11.1% vs. 8.3%, *P* = 0.725).	Aortic expansion is associated with false lumen status, but the difference in reintervention rate did not reach statistical significance.
Vendramin I et al. (2023)	188 patients	Single-center retrospective cohort study	Multivariable Cox regression, inverse probability weighting	Not significant (*P* = 0.11)	Anticoagulation therapy was not associated with reintervention risk (HR = 0.55, *P* = 0.11); the anticoagulation group had a higher rate of patent false lumen (53% vs. 38%, *P* = 0.09) but no increase in reintervention risk.	Diverges from the conclusion of Song SW et al. (2011) that anticoagulation is beneficial for false lumen thrombosis.
Larsson M et al. (2021)	156 patients	Single-center retrospective cohort study	Multivariable logistic regression, Kaplan-Meier	Not significant (*P* = 0.09–0.21)	False lumen status was not significantly associated with survival (*P* = 0.21) or the composite endpoint of survival + freedom from reintervention (*P* = 0.09); 81% of patients had a patent false lumen.	Contrary to the conclusions of most studies, no association between patent false lumen and reintervention was confirmed.

#### Other imaging-related risk factors

**Multiple Intimal Tears (≥ 3)**

One study reported that the presence of ≥ 3 intimal tears in the aortic lumen on early postoperative CTA was significantly associated with an increased risk of aortic-related adverse events (including reintervention), and was identified as an independent risk factor for long-term aortic adverse events after multivariable regression analysis [25].

**False Lumen/True Lumen Ratio (F/T Index) > 1**

Two studies reported that a false lumen-to-true lumen diameter ratio > 1 at the mid-descending thoracic aorta on early postoperative CTA was an independent predictor of late reoperation [7, 26].

**Supra-aortic Branch Vessel Involvement and Distal Anastomotic New Entry (DANE)**

One study showed that preoperative CTA demonstrating dissection involving the supra-aortic branch vessels (e.g., brachiocephalic trunk, left common carotid artery) was an independent risk factor for the development of DANE and was associated with a higher long-term reintervention rate. On postoperative CTA, a new intimal tear appearing within 2 cm of the distal anastomosis significantly increased the risk of distal aortic reintervention [27].(Table 4).

**Table 4:** Other risk factors

Risk factor	Primary study (Sample size)	Study design	Statistical method and adjustment	Significance after multivariable analysis	Replicability / Heterogeneity
Multiple intimal tears (≥ 3)	Kim et al. (2024) *n* = 103	Single-center retrospective cohort	Multivariable Cox regression; decision tree model	Significant (HR 7.12; *p* < 0.001)	Limited evidence, definitions not fully uniform
F/T index > 1	Suzuki et al. (2018) *n* = 278	Single-center retrospective cohort	Multivariable Cox regression	Significant (HR 2.5; *p* = 0.009)	Good replicability
F/T index > 1	Igarashi et al. *n* = 78	Single-center retrospective cohort	Multivariable Cox regression	Significant (*p* = 0.005)	Primarily assessed by F/T index, definition relatively uniform
Distal anastomotic new entry (DANE)	Ahmad et al. (2025) *n* = 327	Single-center retrospective cohort	Competing risk model (Fine-Gray); multivariable regression	Significant (sHR 2.28; *p* = 0.005)	Limited evidence, single-center study

### Endovascular reintervention modalities

A total of 8 studies reported endovascular reintervention techniques, with sample sizes ranging from 14 to 155 patients. The majority were single-center retrospective studies; only Lescan et al. (two-center) and Eleshra et al. 2024 (18-center registry study) were multicenter in design [19, 28–34]. The pathologies treated included true lumen stenosis, distal aneurysmal dilation, and visceral malperfusion. Interventional techniques ranged from standard TEVAR to the Candy-Plug (third-generation), STABILISE, false lumen embolization, and dense metal stents, with substantial differences in technical principles and procedural complexity. Furthermore, the definitions of “aortic remodeling” varied across studies (diameter change vs. volume change vs. thrombosis grading), and follow-up durations ranged from 6 months to 34.4 months, affecting the assessment of long-term outcomes. (Table 5).

**Table 5:** Endovascular intervention strategies

Study (First author, year)	Intervention technique	Sample size	Study design	Inter-study heterogeneity	Complete false lumen thrombosis	Aortic remodeling	Major complications
Lescan et al., 2019	Relay NBS non-bare stent TEVAR	93 patients (39 acute, 54 chronic)	Two-center retrospective analysis	Moderate (unified analysis criteria across centers, single stent type)	Postoperative 73% → Follow-up 84% (*P* < 0.0001)	True lumen expansion; aortic diameter decreased at the mid-stent segment (*P* = 0.009)	d-SINE 15%; type IA endoleak 4%
Rohlffs et al., 2017	Candy-Plug (first generation)	18 patients (chronic dissection)	Single-center retrospective analysis	High (single-center, small sample, custom-made device)	Postoperative 15/18 (83%)	Follow-up > 6 months: 7/10 remodeling, 3/10 stable	1 transient spinal cord ischemia; no death or reintervention
Eleshra et al., 2019	Candy-Plug II (second generation)	14 patients (8 chronic, 6 subacute)	Single-center retrospective analysis	High (single-center, small sample, novel device)	Postoperative 12/14 (86%)	Follow-up 8 months: 8/9 remodeling, 1/9 stable	1 death from false lumen rupture
Faure et al., 2021	STABILISE	17 patients (chronic)	Single-center retrospective analysis of a prospectively maintained database	Moderate (relatively standardized technique)	88% (15/17, down to the renal artery level)	Median aortic diameter reduction of 3 mm	1 type I endoleak
Faure et al., 2019	STABILISE (after DeBakey type I repair)	16 patients (acute phase)	Single-center retrospective analysis	Moderate (specific population, same technique)	100% (complete thrombosis in stented segment)	Complete aortic remodeling in all patients	1 type I endoleak requiring reintervention; 8 renal artery stents (12.5%)
Eleshra et al., 2024	Candy-Plug (mixed generations)	155 patients (32 urgent, 123 elective)	Multicenter retrospective registry study (18 centers)	Moderate (multicenter but retrospective, mixed three generations of the device)	Early: urgent 75% vs. elective 78%	Urgent group remodeling 96% (18% shrinkage + 79% stable); elective group remodeling 96% (55% shrinkage + 41% stable)	Urgent group reintervention 19% vs. elective group 5%
Kuo et al., 2023	Abdominal false lumen embolization	24 patients	Single-center retrospective analysis	High (large inter-study variation; authors specifically noted “survivor bias”)	Abdominal complete thrombosis only 12.5% (3/24)	No significant change in thoracic aortic diameter (1.4 ± 5.6 mm); significant increase in abdominal diameter (4.3 ± 3.7 mm, *P* < 0.005)	No intraoperative complications; 1 death from false lumen rupture during follow-up
Karaolanis et al., 2024	Modified Candy-Plug (pmCP)	17 patients	Two-center retrospective analysis	Moderate (two-center but small sample, different stent platforms used)	Early 82%; follow-up 75% complete thrombosis	13% remodeling; 69% stable; 6% enlargement	1 early reintervention; no spinal cord ischemia or cerebrovascular events

## Discussion

Adverse distal aortic remodeling after type A aortic dissection (TAAD) repair is a central issue affecting the long-term prognosis of patients. Although open surgery represented by Sun’s procedure has significantly reduced early mortality from TAAD, more than 65% of patients still have a residual distal dissection postoperatively. Progressive dilation and rupture driven by persistent false lumen perfusion represent the leading cause of death after an initially successful operation [35]. Therefore, a deeper understanding of the risk factors for adverse distal remodeling, the establishment of a scientific evaluation system, and the exploration of effective intervention strategies are of great importance for improving the long-term outcomes of TAAD patients.

### Pathological mechanisms and risk factors for adverse distal remodeling

The essence of adverse distal aortic remodeling is that after closure of the proximal entry tear, residual distal re-entry tears persistently feed the false lumen, preventing complete thrombosis and even causing progressive dilation. The risk factors for this process can be grouped into three dimensions: anatomical factors, patient-related factors, and surgery-related factors.

#### (1) False lumen patency and thrombosis status

False lumen status is the most critical factor influencing distal remodeling. The study by Kimura et al. showed that the postoperative false lumen patency rate was 62%, and patients with a patent false lumen had a hazard ratio (HR) of 4.11 for distal aortic events, whereas patients with a partially thrombosed false lumen had the fastest aortic growth rate (0.35 mm/month), exceeding that of patients with a completely patent false lumen (0.09 mm/month) [20]. Song et al. also confirmed that a partially thrombosed false lumen is an independent predictor of aortic enlargement (*P* = 0.005), aortic-related reoperation (*P* = 0.002), and poor long-term survival (*P* = 0.023) [24]. The pathophysiological mechanism of this phenomenon is that partial thrombosis can obstruct the outflow of the false lumen, leading to elevated intraluminal pressure and increased wall tension; meanwhile, the aortic wall adjacent to the thrombus triggers an inflammatory cascade and neovascularization due to hypoxia, ultimately causing wall weakness and dilation [36].

#### (2) Distal Anastomotic New Entry (DANE)

DANE refers to a newly developed intimal tear at the distal anastomosis that allows blood flow from the true lumen into the false lumen. A systematic review showed that the incidence of DANE after hemiarch replacement can be as high as 70%, and it is an important cause of aortic dilation and poor prognosis [37]. The study by Ahmad et al. confirmed that the 10-year cumulative incidence of distal aortic reoperation was significantly higher in the DANE group than in the non-DANE group (30% vs. 12%, *P* = 0.0001), and DANE was an independent risk factor for distal reoperation (HR = 2.28, *P* = 0.005). Marway et al. further indicated that DANE, rather than residual arch tears, is significantly associated with negative remodeling of the descending aorta and distal intervention; DANE increases the postoperative descending aortic diameter by 6.2 mm and increases the risk of distal intervention by 3.24-fold [27]. The occurrence of DANE is closely related to surgical technique—procedures performed by dedicated aortic surgeons significantly reduce the risk of DANE (OR = 0.29, *P* = 0.05), whereas preoperative supra-aortic branch vessel involvement increases the risk of DANE (OR = 2.42, *P* = 0.002).

#### (3) Aortic diameter and growth rate

Early postoperative aortic diameter is an important predictor of long-term events. Leontyev et al. found that a postoperative descending aortic diameter > 40 mm was an independent predictor of an aortic diameter increase ≥ 10 mm (OR = 5.6, *P* = 0.006) and an independent predictor of aortic-related reoperation (OR = 4.1, *P* = 0.07). The study by Zierer et al. demonstrated that when the aortic diameter was ≥ 50 mm, 37% of follow-up intervals exhibited growth, compared with only 11% when the diameter was < 35 mm (*P* < 0.001) [9]. Kim et al. found that a postoperative maximal aortic diameter ≥ 40 mm on CTA was significantly associated with aortic dissection-related adverse events (HR = 4.90, *P* = 0.001). Biancari et al. (2024), in a large multicenter registry study (ERTAAD) involving 990 patients, showed that preoperative maximal abdominal aortic diameter was an independent predictor of distal aortic reoperation (SHR = 1.041 per 1 mm increase, *P* < 0.001), and when the abdominal aortic diameter exceeded 30 mm, the 10-year cumulative incidence of distal reoperation was significantly higher (12.0% vs. 5.7%, SHR = 2.076) [8].

#### (4) Patient-related risk factors

Marfan syndrome and other connective tissue diseases are important risk factors for adverse distal remodeling. Junyu Wang et al. reported that the 10-year distal reoperation rate after TAAD repair in patients with Marfan syndrome was 22%, compared with 9.3% in non-Marfan patients over the same period [38]. Beckmann et al. reported that the 15-year distal aortic reintervention rate after FET in Marfan patients was as high as 56%. In addition, young age, male sex, and poorly controlled hypertension are also important predictors of distal aortic events [39]. Zierer et al. found that the 10-year freedom from reoperation was only 57% in patients with systolic blood pressure > 140 mmHg, compared with 83% in those with systolic blood pressure < 120 mmHg (*P* = 0.05) [4].

#### (5) Surgery-related factors

The surgical approach has a significant impact on distal remodeling. After hemiarch replacement, the false lumen patency rate is relatively high (62%), whereas total arch replacement combined with frozen elephant trunk (FET) can significantly increase the rate of false lumen thrombosis. However, the study by Ohtsubo et al. suggests that total arch replacement may increase operative mortality and the risk of neurological complications. Furthermore, an unresected proximal entry tear (HR = 2.26, *P* = 0.005) is also an independent risk factor for distal aortic events [40].

### Assessment methods and risk stratification for distal remodeling

#### Risk prediction models

Based on the above risk factors, several studies have attempted to establish risk stratification models. Akita et al. found that a false lumen/true lumen area ratio > 1 (HR = 3.07, *P* < 0.001) and DANE (HR = 2.34, *P* = 0.017) were independent risk factors for distal aortic events; the 10-year freedom from distal events was only 42.3% in the high-risk group, compared with 83.3% in the low-risk group [41]. Kim et al. proposed a decision tree model based on postoperative CTA, classifying patients with a maximal aortic diameter ≥ 40 mm and ≥ 3 intimal tears as the high-risk group; their 3-year event-free survival was only 41.7%, compared with 90.9% in the low-risk group [40].

### Treatment strategies to promote distal aortic remodeling

#### (1) Frozen Elephant Trunk (FET) Technique

By combining total arch replacement and descending aortic stent-graft placement in a single stage, FET can effectively seal tears in the arch and proximal descending aorta, promoting false lumen thrombosis [42]. Borghese et al. reported a false lumen thrombosis rate of 95.1% after FET, but the remodeling effect was mainly confined to the thoracic aortic segment (Ishimaru zones 1–4, *P* < 0.001) and was limited in the abdominal aortic segment (zones 5–7) [43]. Berger et al. found that in chronic dissection patients, the true lumen diameter increased from 15 mm to 28 mm (*P* = 0.001) and the false lumen diameter decreased from 40 mm to 32 mm (*P* = 0.026) at 2 years after FET. The clinical advantage of FET also lies in providing an ideal proximal landing zone for subsequent interventions, but the distal aortic reintervention rate remains 17.1%–28% [42].

#### (2) PETTICOAT and STABILISE Techniques

The PETTICOAT (Provisional Extension To Induce Complete Attachment) technique extends a bare-metal stent distal to the covered stent graft into the abdominal aorta, directly expanding the abdominal true lumen and promoting false lumen thrombosis. Huang et al. found that the abdominal true lumen volume increased by 46.6 mL at 1 year in the PETTICOAT group, which was significantly higher than the 30.1 mL increase in the non-PETTICOAT group (*P* = 0.001), and the false lumen thrombosis rate was also higher (54.0% vs. 25.5%, *P* = 0.016) [44]. The STABILISE (Stent-Assisted Balloon-Induced Intimal Disruption and Relamination) technique achieves more complete aortic remodeling in chronic dissection by creating a controlled intimal disruption and reapposition through balloon dilation of a bare-metal stent. Faure et al. reported 17 cases of chronic descending aortic dissection treated with STABILISE, with 88% achieving complete false lumen thrombosis in the treated thoracoabdominal aortic segment. It should be noted, however, that the long-term efficacy of STABILISE still requires validation with larger sample sizes and longer follow-up [32].

#### (3) False Lumen Embolization Techniques (Candy-Plug, etc.)

For patients in whom false lumen thrombosis cannot be achieved by conventional TEVAR, the Candy-Plug technique provides a solution for direct false lumen occlusion. An international multicenter registry study showed that in 155 patients with chronic dissection treated with the Candy-Plug technique, the technical success rate was 100%, the clinical success rate was 89%, and the early complete false lumen occlusion rate reached 77%. The technique has proven feasible in both urgent and elective patients, although early mortality was higher in the urgent group (13%) than in the elective group (1%, *P* = 0.021), which may be related to the patients’ generally poorer condition [30].

#### (4) Transcatheter Electrosurgical Septotomy (TES)

TES is a novel adjunctive technique developed in recent years that uses electrosurgical energy to lacerate the intimal flap, unifying the true and false lumens and creating a larger working space for branch vessel reconstruction and stent delivery. Kanamori et al. reported 36 cases of chronic dissection in which the TES technical success rate reached 92%; the postoperative aortic luminal diameter increased from 13.2 mm to 28.4 mm (*P* < 0.001) without aortic rupture or target vessel occlusion. The technique is safe and effective in chronic dissection but requires caution in acute dissection (technical success rate only 33%) [45].

#### (5) Novel Devices: Dense Mesh Stent and Ascyrus Medical Dissection Stent (AMDS)

The dense mesh stent, with an optimized porosity (approximately 70%), provides uniform radial support, promotes intimal flap apposition, and preserves branch vessel flow. Li et al. reported that in 25 patients with residual dissection, the true lumen volume increased by 141.26% and the false lumen volume decreased by 79.32% at 30 days postoperatively, with all visceral branches remaining patent [46]. The AMDS, a novel hybrid arch branch stent-graft, stabilizes the true lumen of the aortic arch and proximal descending aorta. White et al. found that the DANE incidence was only 11.8% in the AMDS group, significantly lower than the 43.3% in the hemiarch-only group (*P* = 0.002), and the AMDS was superior in terms of false lumen thrombosis in the aortic arch (*P* = 0.029) and proximal descending aorta (*P* = 0.031) [47].

### Summary and future directions

Adverse distal aortic remodeling after TAAD repair is a complex issue driven by multiple factors. False lumen patency, DANE, aortic diameter, connective tissue disease, and surgical approach are all important factors influencing distal remodeling. Morphological assessment and risk stratification based on postoperative CTA help identify high-risk patients and guide individualized follow-up and intervention strategies.

At the therapeutic level, new techniques such as FET, PETTICOAT, STABILISE, Candy-Plug, TES, and AMDS each have their own advantages, offering more options to promote distal aortic remodeling. However, most current studies are single-center, retrospective in design, and have limited follow-up; their long-term efficacy still requires validation. Future research directions should include: [1] establishing a standardized evaluation system for distal remodeling; [2] conducting multicenter prospective studies to verify the superiority of different treatment strategies; [3] exploring the application of novel technologies such as radiomics and hemodynamic assessment in risk prediction; and [4] developing individualized strategies for the timing and modality of intervention based on different risk factors.

## Conclusion

Adverse distal aortic remodeling after TAAD repair is synergistically influenced by imaging-related factors, underlying clinical diseases, and surgery-related factors, with false lumen status serving as a core prognostic indicator throughout the entire course. Clinically, individualized follow-up protocols need to be formulated based on risk stratification, and intensified surveillance and early intervention are warranted for specific high-risk populations to improve long-term patient outcomes.

## Data Availability

No datasets were generated or analysed during the current study.
